# Intra-abdominal rectal perforation post-haemorrhoidal artery ligation operation and converted Ligasure open haemorrhoidectomy

**DOI:** 10.1093/jscr/rjab289

**Published:** 2021-07-09

**Authors:** Natalie Simon, Noshin Saiyara, Hyun-Kyung Kim, Yuksel Gercek

**Affiliations:** Bedford Hospital, Bedford Hospital NHS Foundation Trust, Bedford, UK; Cambridge Academic Training Office, Cambridge University, Cambridge University Hospitals NHS Foundation Trust, Cambridge, UK; Bedford Hospital, Bedford Hospital NHS Foundation Trust, Bedford, UK; Bedford Hospital, Bedford Hospital NHS Foundation Trust, Bedford, UK; Bedford Hospital, Bedford Hospital NHS Foundation Trust, Bedford, UK

## Abstract

We report an unfortunate case of rectal perforation and subsequent peritonitis in a 74-year-old lady who underwent haemorrhoidal artery ligation in order to treat complex large external and internal haemorrhoids. Serious complications following haemorrhoid surgery are rare and this is the first documented case of delayed intra-abdominal rectal perforation following a haemorrhoid artery ligation operation and converted Ligasure open haemorrhoidectomy.

## INTRODUCTION

Haemorrhoids are described as enlarged anal vascular cushions that typically cause discomfort and rectal bleeding on defecation. Management is conservative or surgical, depending on the severity of symptoms and degree of prolapse. Surgical options include traditional haemorrhoidectomy (open or closed) with or without energy devices, stapled haemorrhoidopexy or Doppler-guided artery ligation such as the haemorrhoid artery ligation operation (HALO). Complications post-haemorrhoid surgery are rare, with serious complications such as rectal perforation and subsequent peritonitis scarce in published cases. We present a case of rectal perforation to the intra-abdominal cavity in a 74-year-old lady, identified 7 days after HALO and subsequent converted open Ligasure haemorrhoidectomy.

## CASE REPORT

A 74-year-old lady was referred to our department complaining of fresh rectal bleeding on defecation for many years. Her past medical history included primary biliary cirrhosis and oesophageal varices, for which she was medicated with rifaximin, ursodeoxycholic acid, spironolactone and omeprazole. Her operative history included a laparoscopic cholecystectomy and a hysterectomy. On examination, her abdomen was non-tender with no palpable masses. Proctoscopy and rigid sigmoidoscopy revealed the presence of two large external haemorrhoids with three internal haemorrhoids in the 3, 7 and 11 o’clock position. The rectal mucosa looked normal at 20 cm of visualization.

It is of note that, in the past, she had presented to another surgeon with symptomatic haemorrhoids. At this time, it was felt that her risk for operative treatment was too high due to cirrhosis. On this occasion, however, operative treatment was deemed necessary and the patient subsequently underwent HALO. Examination under anaesthesia and sigmoidoscopy was performed intra-operatively. Large fourth degree prolapsing haemorrhoids with a large intra-rectal venous component and five large internal haemorrhoids were identified. Two large arteriovenous fistulae were seen at 7 and 11 o’clock. Unfortunately, attempts at suturing caused major bleeding of mixed venous and arterial type. This was not controlled by simple suturing and, therefore, the procedure was converted to open Ligasure haemorrhoidectomy, performed with the aid of a Park’s retractor. A post-operative sigmoidoscopy revealed no damage to surrounding organs or further bleeding. She was discharged after 48 h of inpatient observation.

Unfortunately, the patient re-presented to the emergency department at 5 days post-discharge complaining of abdominal pain and constipation. On examination, umbilical tenderness with guarding was elicited and bowel sounds were present. Remarkable admission laboratory tests included an elevated C-reactive protein (234.6), acute kidney injury stage 1 (creatinine of 119, urea of 13.6), deranged anticoagulation (INR of 1.6 and patient not on anticoagulation) and hyponatraemia (sodium of 124). A venous blood gas revealed a lactate of 3.5 with a normal pH and bicarbonate level. Haemoglobin and platelet counts were normal. A computerized tomography (CT) abdomen and pelvis scan showed a large amount of pneumoperitoneum and ascites. After initial resuscitation, urgent laparoscopy was performed. After laparoscopic insertion, pus and peritonitis was seen in all quadrants and early conversion to laparotomy was done. Many adhesions and a large faecal collection were seen upon entering the abdominal cavity. A 2-cm rectal perforation with clean edges was discovered at the left anterior side of the rectum at the level of the peritoneal reflection. The sigmoid and upper rectum was mobilized. The bowel was divided close to the perforation. An end colostomy was brought out. Abdominal lavage was done with copious amounts of saline and the distal rectum was washed through the anus with saline until the fluid returned clear. Two Robinson drains were inserted, and the abdomen was closed. The patient was subsequently admitted to the intensive care unit (ICU) for close observation. Initially, her vital readings were stable, and her stoma was functioning normally. She was extubated 48 h post-laparotomy. Unfortunately, whilst in ICU, her inflammatory markers continued to rise, and her renal and respiratory function deteriorated. She was deemed to be in multi-organ failure and sadly passed away 12 days post-laparotomy.

## DISCUSSION

Haemorrhoids are defined as a pathological enlargement of the anal vascular cushions and are classified according to extent of prolapse; first degree haemorrhoids are within the rectum, second degree haemorrhoids prolapse externally on defecation while spontaneously reducing, third degree haemorrhoids require manual reduction once prolapsed and fourth degree haemorrhoids cannot be reduced. Conservative management or banding is reserved for lesser degree haemorrhoids, whereas HALO, stapled haemorrhoidectomy or the conventional Milligan Morgan open haemorrhoidectomy procedure are necessary to repair third and fourth degree haemorrhoids. The conventional approach is most often used for third and fourth degree haemorrhoids, while HALO is efficacious for second and third degree haemorrhoids [1, 2]. The HALO procedure utilizes a modified proctoscope containing a Doppler probe to detect haemorrhoidal arteries and dictate where they should be ligated with sutures [1]. Ligasure haemorrhoidectomy works by applying a precise amount of bipolar energy and pressure to fuse the collagen and elastin in the vessel walls and can be used to excise haemorrhoidal tissue [3].

Severe post-haemorrhoidectomy complications such as rectal perforation and peritonitis are rare [4]. In published literature, cases of rectal perforation most commonly happen after stapled haemorrhoidopexy [3, 5–7]. Procedure for prolapsed haemorrhoids (stapled haemorrhoidopexy) has been seen to cause rectal free perforation, leading to pneumoperitoneum and peritonitis, secondary to staple dehiscence [7]. Staple dehiscence is to blame for resultant perforation in a number of other cases [8–11]. Excessive bleeding leading to perforation due to increased pressure from a rectal haematoma is to blame for another case of post-stapled haemorrhoidopexy peritonitis, requiring low anterior resection for repair [8, 12]. While stapled haemorrhoidopexy is responsible for the majority of reported rectal perforation cases, there are scarce reports of perforation following HALO and open haemorrhoidectomy procedures [13–15]. Following transanal dearterialization (THD), rectosigmoid junction perforation causing left lateral extraperitoneal inflammation and upper rectal perforation causing circumferential mesorectal inflammation and left lateral collection has been documented [14]. Further focal perforation resulting in sepsis without peritonitis has been reported 48 h post-HALO [13].

We cannot explain why rectal perforation occurred in this case. Acknowledging that post-operative sigmoidoscopy was unremarkable, it is likely that this was a delayed perforation. A thermal injury from the Ligasure device is possible with delayed perforation. Delayed perforations have been documented following endoscopic colorectal procedures and this has been termed coagulation syndrome or transmural burn, resulting in localized peritonitis and serosa breakdown [16]. An alternative explanation is that this was traction injury caused by manipulation and retraction in a patient under general anaesthetic with a deep recto-vesical pouch. Furthermore, this patient had significant pre-operative risk factors for portal hypertension and suffered excessive intraoperative bleeding owing to profound arteriovenous fistulae. Post-operative rectal wall haematoma and delayed bleeding should be also considered a causative factor. It is likely that the aetiology of this complication is multi-factorial with the exact cause not able to be established.

As far as we are aware, this is the first documented case of delayed intra-abdominal rectal perforation following HALO and converted Ligasure open haemorrhoidectomy. To determine this, PubMed online library and MEDLINE database were searched from 1996 to 10 January 2021 with search terms ‘rectal perforation’ and ‘haemorrhoidectomy’.

We encourage surgeons to be cognizant of delayed intra-abdominal perforation as a potential complication of Doppler-guided THD/HALO and Ligasure-assisted open haemorrhoidectomy. Follow-up CT scanning in the post-operative period may be beneficial in patients with intra-operative complications. Moreover, consideration of pre-operative optimization in patients with portal hypertension is pertinent. Pre-operative transjugular intrahepatic porto-systemic shunting or medical management should be regarded [17].

## CONFLICT OF INTEREST STATEMENT

None declared.

## FUNDING

None.

## References

[1] Miah M, Centea D, Michael G, Husain N, Virlos I, Al Saramigy M. Hemorrhoidal artery ligation operations-recto-anal repair (HALO-RAR) procedure for recurrent haemorrhoids: excellent patient satisfaction. Cureus 2020;12:e7944. 10.7759/cureus.7944 PMC7266563 32499984

[2] Lohsiriwat V. Treatment of hemorrhoids: a coloproctologist’s view. World J Gastroenterol 2015;21:9245. 10.3748/wjg.v21.i31.9245 PMC4541377 26309351

[3] Brown SR. Haemorrhoids: an update on management. Ther Adv Chronic Dis. 2017;8:141–7. 28989595 10.1177/2040622317713957 PMC5624348

[4] Klang E, Sobeh T, Amitai MM, Apter S, Barash Y, Tau N. Post hemorrhoidectomy complications: CT imaging findings. Clin Imaging 2020;60:2016–221. 10.1016/j.clinimag.2019.12.015 31927497

[5] Ripetti V, Caricato M, Arullani A. Rectal perforation, retropneumoperitoneum, and pneumomediastinum after stapling procedure for prolapsed hemorrhoids: Report of a case and subsequent considerations. Dis Colon Rectum 2002;45:268–70. 10.1007/s10350-004-6159-3 11852343

[6] Wong L-Y, Jiang J-K, Chang S-C, Lin J-K. Rectal perforation: a life-threatening complication of stapled hemorrhoidectomy. Dis Colon Rectum 2003;46:116–7. 12544531 10.1007/s10350-004-6505-5

[7] Ryu S, Bae BN. Rectal free perforation after stapled hemorrhoidopexy: a case report of laparoscopic peritoneal lavage and repair without stoma. Int J Surg Case Rep 2017;30:40–2. 10.1016/j.ijscr.2016.11.031 PMC5133467 27902953

[8] Faucheron JL, Voirin D, Abba J. Rectal perforation with life-threatening peritonitis following stapled haemorrhoidopexy. Br J Surg 2012;99:746–53. 10.1002/bjs.7833 22418745

[9] Pessaux P, Lermite E, Tuech JJ, Brehant O, Regenet N, Arnaud JP. Pelvic sepsis after stapled hemorrhoidectomy. J Am Coll Surg 2004;199:824–5. 15501126 10.1016/j.jamcollsurg.2004.04.026

[10] Karadeniz Cakmak G, Irkorucu O, Ucan BH, Karakaya K. Fournier’s gangrene after open hemorrhoidectomy without a predisposing factor: report of a case and review of the literature. Case Rep Gastroenterol 2009;3:147–55. 10.1159/000218091 PMC2988949 21103267

[11] Maw A, Eu KW, Seow-Choen F. Retroperitoneal sepsis complicating stapled hemorrhoidectomy: report of a case and review of the literature. Dis Colon Rectum 2002;45:826–8. 10.1007/s10350-004-6304-z 12072637

[12] Blouhos K, Vasiliadis K, Tsalis K, Botsios D, Vrakas X. Uncontrollable intra-abdominal bleeding necessitating low anterior resection of the rectum after stapled hemorrhoidopexy: report of a case. Surg Today 2007;37:254–7. 10.1007/s00595-006-3363-x 17342370

[13] Greensmith S, Ip B, Vujovic Z. Rectal perforation secondary to transanal haemorrhoidal dearterialisation. Ann R Coll Surg Engl 2017;99:154–5. 10.1308/rcsann.2017.0059 PMC5449713 28462643

[14] Sturiale A, Cafaro D, Fabiani B, Ferro U, Naldini G. Rectal perforation after Doppler-guided hemorrhoidal dearterialization treated with diverting sigmoidostomy. Tech Coloproctol 2018;22:553–4. 30062433 10.1007/s10151-018-1824-z

[15] Berstock JR, Bunni J, Torrie AP. The squelching hip: a sign of life-threatening sepsis following haemorrhoidectomy. Ann R Coll Surg Engl 2010;92:39–41. 10.1308/147870810X12699662980394 PMC5696956 20529481

[16] Hirasawa K, Sato C, Makazu M, Kaneko H, Kobayashi R, Kokawa A, et al. Coagulation syndrome: delayed perforation after colorectal endoscopic treatments. World J Gastrointest Endosc 2015;7:1055–61. 26380051 10.4253/wjge.v7.i12.1055 PMC4564832

[17] Galati G, De Vincentis A, Ripetti V, La Vaccara V, Vespasiani-Gentilucci U, Mazzarelli C, et al. Haemorrhoidal disease in severe portal hypertension: a combined approach with transjugular intrahepatic portosystemic shunt (TIPS) and transanal haemorrhoidal dearterialization (THD). Arch Med Sci 2014;1:195–6. 10.5114/aoms.2014.40746 PMC3953988 24701234

